# Berry curvature driven transverse thermoelectric generation in topological magnets

**DOI:** 10.1080/14686996.2025.2554047

**Published:** 2025-09-16

**Authors:** Akito Sakai, Satoru Nakatsuji

**Affiliations:** a Department of Physics, University of Tokyo, Bunkyo-ku, Japan; b Institute for Solid State Physics, University of Tokyo, Chiba, Japan; c Institute for Quantum Matter and Department of Physics and Astronomy, Johns Hopkins University, Baltimore, MD, USA; d Trans-Scale Quantum Science Institute, University of Tokyo, Bunkyo, Japan; e Canadian Institute for Advanced Research (CIFAR), Toronto, ON, Canada

**Keywords:** Topological magnets, Weyl semimetals, nodal line semimetals, anomalous Nernst effect

## Abstract

Topological magnets such as magnetic Weyl and nodal-line semimetals possess topologically non-trivial band structures in magnetically ordered states. In this class of materials, the Berry curvature in momentum space can be significantly enhanced, resulting in thermoelectric responses that exceed empirical scaling laws based on magnetization. Such large transverse thermoelectric effects enable flexible thin-film-based lateral device structures, leading to novel energy-harvesting technologies and sensors beneficial for Internet of Things (IoT) sensors and wearable devices. In this review, we outline recent progress in the study of the large transverse thermoelectric effects in topological magnets.

## IMPACT STATEMENT

We review recently developed transverse thermoelectric generation technology using topological magnets and cover its novel mechanism and applications.

## GRAPHICAL ABSTRACT

**Figure:**
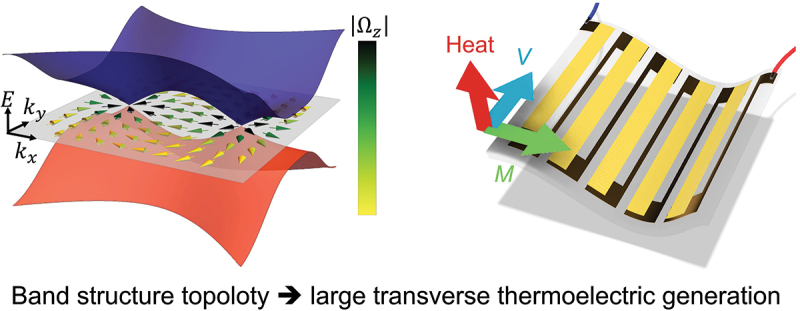

## 1. Introduction

**Figure 1.**
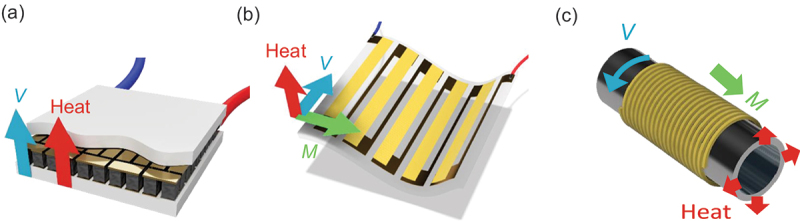
Schematic figures of thermoelectric devices based on the longitudinal (a) and transverse (b, c) thermoelectric effects (Adapted from [4]). In the Seebeck-effect-based (Peltier) device, a large number of p- and n-type semiconductor pillars are necessary to enhance the output voltage. In contrast, ANE-based devices can achieve increased output voltage in a thin-film form, which is suitable for covering large areas and curved heat sources.

Thermoelectric effects, such as the Seebeck effect, directly convert heat into electricity (and vice versa) in solids and have been extensively studied for applications in heat exchange, waste heat utilization, and as power sources for IoT sensors [1–3]. In particular, Peltier devices based on the Seebeck effect are now widely used as wearable coolers (Figure 1). Recently, the transverse thermoelectric conversion in magnets, known as the anomalous Nernst effect (ANE), has attracted much attention because it allows a lateral configuration of the thermoelectric modules that can efficiently cover heat sources (Figure 1) [5–8]. Extensive studies on the transverse thermodynamic effects have identified topological magnets as a key material class exhibiting a large ANE [8], which we review in this article.

Recent developments in condensed matter physics have revealed that non-trivial topological band structures near the Fermi energy *ε*_F_ are important for understanding the unique properties of materials [9–12]. Among them, topological magnets such as magnetic Weyl semimetals and nodal-line semimetals have been found to exhibit large transverse responses such as the ANE and anomalous Hall effect (AHE) [4,6,8,13–28] owing to the non-vanishing momentum-space (*k*-space) Berry curvature. Various research groups have demonstrated ANE-based thermoelectric devices [5,7,29–34], which may be useful for energy harvesting from waste heat, IoT sensors, and wearable devices.

In this article, we review the recent advances in research and development of transverse thermoelectric generation in topological magnets. We first introduce the ANE as the transverse thermoelectric generation for magnetic materials (Section 2). Then, we briefly illustrate the theoretical framework for the Berry curvature–driven intrinsic ANE in topological magnets (Sections 3–5). We also review the experimental observations for ANE in various typical topological magnets (Section 6). Finally, we provide a summary and perspective (Section 7).

## 2. Anomalous Nernst effect – transverse thermoelectric effect in magnetic mateirals

Generally in solids, the electric current density **J**_*c*_, is proportional to the electric field **E**, **J**_*c*_ = *σ***E**, known as Ohm’s law. Similarly, the heat current density **J**_*q*_ is proportional to the temperature gradient ∇*T*, **J**_*q*_ = *κ*(−∇*T*), known as Fourier’s law. Here, *σ* and *κ* are electric and thermal conductivity tensors. In metals and semiconductors, carriers (electrons or holes) transport both electric and heat current. This means that **E** (∇*T*) also generates **J**_*q*_ (**J**_*c*_). Thus, the above equations are generalized in the linear response regime as,

(1) $$J_{c}=\sigma E+\alpha (-\mathrm{\nabla T}),$$

(2) $$J_{q}=\Pi E+\kappa (-\nabla T).$$

The newly added cross-correlated terms represent the thermoelectric effects with thermoelectric tensors *α* and Π. According to the Onsager reciprocal relations, *α* and Π are not independent and satisfy Π = *αT* [35–37]. Besides, the Onsager reciprocal relations also hold for the off-diagonal terms of the conductivity tensors,

(3) $$\begin{array}{c} \sigma_{\mathrm{ij}}\left(B\right)=\sigma_{\mathrm{ji}}\left(-B\right),\alpha_{\mathrm{ij}}\left(B\right)=\alpha_{\mathrm{ji}}\left(-B\right),\kappa_{\mathrm{ij}}\left(B\right)=\kappa_{\mathrm{ji}}\left(-B\right), \end{array}$$

where *σ*_*ji*_, *α*_*ji*_ and *κ*_*ji*_ are, respectively, called Hall conductivity, transverse thermoelectric conductivity, and thermal Hall conductivity, and *B* is the magnetic field. The Eq. (3) implies *σ*_*ji*_ = *α*_*ji*_ = *κ*_*ji*_ = 0 when both magnetic field *B* and magnetization *M* are zero (*B* = *M* = 0).

The Onsager reciprocal relations are fundamental for irreversible transport processes originating from microscopic time-reversal symmetry. Thus, they are never violated as long as the system satisfies their precondition; the states at +*B* and −*B* are equivalent to each other after the time-reversal operation. In other words, the relations, such as *σ*_*ij*_(*B*) ≠ *σ*_*ji*_(−*B*),*α*_*ij*_(*B*) ≠ *α*_*ji*_(−*B*), may occur in hysteretic regimes due to magnetic domains or exchange bias. Additionally, *non*-Onsager quantities such as the Nernst coefficient *S*_*ij*_ (defined as Eq. (8) below) do not obey this rule. Thus, *S*_*ij*_(*B*) is not necessarily equal to *S*_*ji*_(−*B*) as observed in YbMnBi_2_ [25,26]. (These two cases should not be called ‘violations’ of the Onsager relations since they simply fall outside the scope of the principles.)

### 2.1. Anomalous Hall effect

The electrical resistivity *ρ* is the inverse of *σ*, *ρ* = *σ*^−1^, and *xx* and *xy* components are given by

(4) $$\sigma_{\mathrm{xx}}=\frac{\rho_{\mathrm{yy}}}{\rho_{\mathrm{xx}}\rho_{\mathrm{yy}}-\rho_{\mathrm{xy}}\rho_{\mathrm{yx}}},\sigma_{\mathrm{xy}}=-\frac{\rho_{\mathrm{xy}}}{\rho_{\mathrm{xx}}\rho_{\mathrm{yy}}-\rho_{\mathrm{xy}}\rho_{\mathrm{yx}}},$$

where we consider the net flow of carriers along the *xy* plane, **J**_*c*_ ⊥ **z** ∥ **B**. Thus, we get *σ*_*xx*_  ∼  1*/ρ*_*xx*_, *σ*_*yx*_ = −*σ*_*xy*_ ∼ −*ρ*_*yx*_*/ρ*^2^_*xx*_ for conventional isotropic 3D metals where *ρ*_*xx*_ ∼ *ρ*_*yy*_ ≫ |*ρ*_*yx*_| and *ρ*_*xy*_(*B*) = −*ρ*_*yx*_(*B*). Experimentally, we measure *ρ*_*xx*_ = *E*_*x*_/*J*_*x*_, and *ρ*_*yx*_ = *E*_*y*_*/J*_*x*_ and obtain *σ*_*xx*_ and *σ*_*xy*_, where *J*_*i*_ and *E*_*i*_ are the applied current density and measured electric field along *i*-direction, respectively.

Historically, Edwin Hall first discovered the Hall effect in nonmagnetic metals and ferromagnets [38,39]. Subsequent studies have established the empirical relation

(5) $$\begin{array}{c} \rho_{\mathrm{yx}}=R_{0}B+R_{s}M, \end{array}$$

where *R*_0_ and *R*_*s*_ are the ordinary Hall and anomalous Hall coefficients, respectively [40]. The first term is the ordinary Hall effect (OHE), related to the carrier density *n* and charge *q* as *R*_0_ = *B/*(*nq*). The second term represents the anomalous Hall effect (AHE), which typically dominates in ferromagnetic states. This has been intuitively interpreted as the consequence of the Lorentz force due to *M*, and thus it was long believed that AHE should not occur in antiferromagnets, where *M* is vanishingly small.

However, this empirical relation (Eq. 5) and the corresponding intuitive interpretation have turned out to be inaccurate; they neglect contributions from Berry curvatures in both real and momentum space. The real-space Berry curvature originates, for example, from skyrmions in noncentrosymmetric magnets [41–47]. The *k*-space Berry curvature leads to the intrinsic AHE, which is distinct from the extrinsic AHE caused by disorder-related mechanisms such as skew scattering and side jump. Importantly, the intrinsic AHE is not necessarily proportional to *M* and can be significant in noncollinear and noncoplanar antiferromagnets (AFM) [8,28,48–50] and even finite in the collinear antiferromagnetic known as altermagnets [51–54]. In Section 4, we discuss the intrinsic (*k*-space Berry curvature driven) AHE in detail and explain how large AHE signals can arise in both ferromagnets and antiferromagnets.

### 2.2. Anomalous Nernst effect

The longitudinal thermoelectric effect, the Seebeck coefficient *S*_*ii*_, is obtained by setting **J**_*c*_ = 0,∇*T* ∥ **x** and *σ*_*ij*_ = *α*_*ij*_ = 0 in Eq. (1),

(6) $$S_{\mathrm{xx}}\equiv \frac{E_{x}}{-\left(\mathrm{\partial T}/\mathrm{\partial x}\right)}=\frac{V}{\Delta T}=\frac{\alpha_{\mathrm{xx}}}{\sigma_{\mathrm{xx}}}.$$

**Figure 2.**
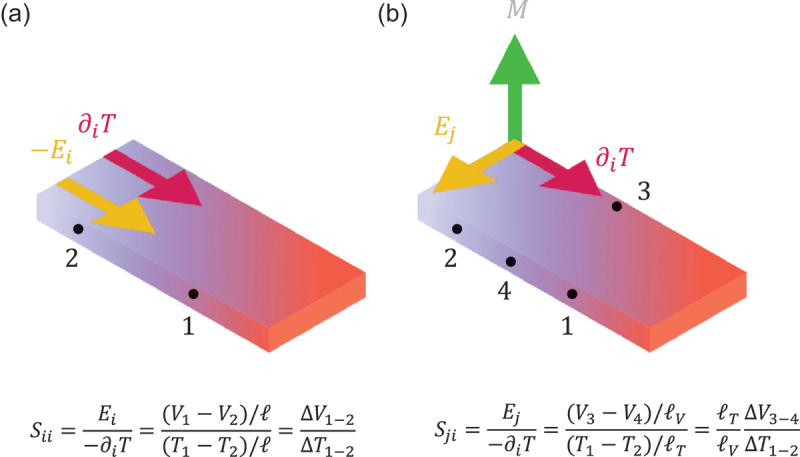
Schematic figures illustrating the Seebeck effect (a) and ANE (b). For the Seebeck coefficient *S*_*ii*_, the sample length *ℓ* appears in both numerator and denominator and cancels out. As a result, *S*_*ii*_ is determined solely by the ratio of voltage to temperature difference ∆*V*_1 − 2_* /* ∆*T*_1 − 2_. In contrast, the sample dimension remains for the anomalous Nernst coefficient *S*_*ij*_ as *S*_*ij*_ = (*ℓ*_*T*_*/ℓ*_*V*_)(∆*V*_3−4_*/*∆*T*_1−2_), where *ℓ*_*T*_ = *ℓ*_1−2_ and *ℓ*_*V*_ = *ℓ*_3 − 4_ respectively represent length between thermometers along the heat current direction and length between transverse voltage terminals. This means that the ANE voltage ∆*V*_3 − 4_ increases by reducing *ℓ*_*T*_ and increasing *ℓ*_*V*_ , as realized in thin-film ANE-based thermoelectric modules (see Figure 1(b,c)).

Note that the sample length cancels out and does not appear in Eq. (6) (see Figure 2(a)).

In contrast, the transverse thermoelectric effect (Nernst effect) can be finite by breaking the time-reversal symmetry, i.e. *B* ≠0 or *M* ≠0. Similar to the Hall effect, *B*-linear and *M*-linear terms are called ordinary Nernst effect (ONE) and ANE, respectively.

By setting $J_{c}=0,\nabla T∥x$ in Eq. (1), we get

(7) $$\alpha_{\mathrm{yx}}=\sigma_{\mathrm{yx}}S_{\mathrm{xx}}+\sigma_{\mathrm{yy}}S_{\mathrm{yx}}$$

where the Nernst coefficient $S_{\mathrm{yx}}$ is defined as

(8) $$S_{\mathrm{yx}}\;\equiv \;\frac{E_{y}}{-\left(\mathrm{\partial T}/\mathrm{\partial x}\right)}=\frac{\ell_{T}}{\ell_{V}}\frac{V_{y}}{\Delta T}.$$

Interestingly, the Nernst voltage *V*_*y*_ is proportional not only to the temperature difference ∆*T* but also to the sample geometry *ℓ*_*V*_ */ℓ*_*T*_ , where *ℓ*_*V*_ and *ℓ*_*T*_ respectively represent distances between voltage and temperature terminals (Figure 2(b)). Thus, one can enhance *V*_*y*_ in thin-film thermopile structure by reducing *ℓ*_*T*_ and increasing *ℓ*_*V*_ (Figure 1(b)), as demonstrated by various experiments [5–7,30,31,33,55]. Eq. (7) immediately provides

(9) $$-S_{\mathrm{yx}}=-\alpha_{\mathrm{yx}}\rho_{\mathrm{yy}}+\sigma_{\mathrm{yx}}\rho_{\mathrm{yy}}S_{\mathrm{xx}}\;\equiv \;S_{1}+S_{2}$$

Here, *S*_1_ and *S*_2_ respectively indicate the contributions directly from *α*_*yx*_ and longitudinal thermoelectric effect *S*_*xx*_ converted to the transverse direction through *σ*_*xy*_. Thus, in addition to large |*α*_*yx*_|, these two contributions should be constructive (namely sgn (−*α*_*yx*_) = sgn (*σ*_*yx*_*S*_*xx*_)) for enhancing |*S*_*yx*_|.

## 3. Berry phase, Berry connection, and Berry curvature

Topology in condensed matter physics appears from the Hilbert space geometry [56]. For simplicity, let us assume a nondegenerate complete orthonormal system $\left\{\;\left|u_{n}\right\rangle \right\}$

(10) $$H\left(R\right)\;\left|u_{n}\left(R\right)\right\rangle =E_{n}\left(R\right)\;\left|u_{n}\left(R\right)\right\rangle ,$$

where $E_{n}$ is the eigenenergy for the n-th eigen state $\left|u_{n}\right\rangle$, and R(t) is a parameter for the Hamiltonian $H\left(R\right)$ such as the wave vector k and real space position r. Then, the time-evolution for the initial state $\left|\psi (t)\right\rangle =\sum_{n}c_{n}\left|u_{n}(R)\right\rangle$ obtained from the time-dependent schrödinger equation $i\hbar \frac{d}{\mathrm{dt}}\left|\psi (t)\right\rangle =H\left(R\right)\left|\psi (t)\right\rangle$ is

(11) $$i\hbar \frac{d}{\mathrm{dt}}c_{n}+i\hbar c_{n}\;\left\langle u_{n}|\frac{d}{\mathrm{dt}}|u_{n}\right\rangle +i\hbar \sum_{m\neq n}\frac{\left\langle u_{n}|\frac{d}{\mathrm{dt}}H|u_{m}\right\rangle }{E_{m}-E_{n}}c_{m}=c_{n}E_{n}.$$

On the other hand, the time derivative of Eq. (10) gives $\left\langle u_{n}|\frac{d}{\mathrm{dt}}|u_{m}\right\rangle =\frac{\left\langle u_{n}|\frac{d}{\mathrm{dt}}H|u_{m}\right\rangle }{E_{m}-E_{n}}$ for n≠m. This indicates the last term on the left-hand side of Eq. (11) is negligible compared to the diagonal term $\left\langle u_{n}|\frac{d}{\mathrm{dt}}|u_{n}\right\rangle$ in the adiabatic approximation, where the time-evolved state stays at the initial eigenstate with a certain phase difference ϕ; $\left|u_{n}(R(t))\right\rangle ∼\left|u_{n}(R(0))\right\rangle \exp(\mathrm{i\phi})$. Solving Eq. (11), we obtain

(12) $$c_{n}(t)=c_{n}(0)\exp(i\theta_{n}(R))\exp(i\gamma_{n}(R)),\;$$

where$\;\theta_{n}(R)\;\equiv -{\frac{1}{h}}_{R_{0}}^{R}E_{n}(R\prime )\mathrm{dR}\prime \text{and}\gamma_{\text{n}}(R)\;\equiv \text{ i}R_{\text{0}}R$ $\left\langle u_{n}(R{\;}^{\prime })|\frac{\partial }{\partial R{\;}^{\prime }}|u_{n}(R{\;}^{\prime })\right\rangle dR{\;}^{\prime }$ are known as the dynamical and geometrical phases, respectively. The former is the conventional term originating from the time evolution of the eigenstate. The latter is the topological term depending on the path C from $R_{0}\;\mathrm{to}\;R$. While the above $\gamma_{n}(R)$is gauge-dependent and not a physical observable, the closed-loop integral so-called the Berry phase

(13) $$\gamma_{n}\left(C\right)\;\equiv \underset{C}{\oint }A_{n}\left(R^{\prime }\right)dR^{\prime }=\underset{S}{\int }\Omega_{n}\left(R^{\prime }\right)dS^{\prime }$$

becomes gauge-independent. Here,

(14) $$A_{n}(R)\;\equiv \;i\;\left\langle u_{n}(R)|\nabla_{R}|u_{n}(R)\right\rangle$$

and

(15) $$\Omega_{n}(R{\;}^{\prime })\;\equiv \;\nabla_{R}\times A_{n}(R)$$

are the Berry connection and Berry curvature, respectively. In real space, $\gamma_{n}(C),A_{n}(r),\Omega_{n}(r)$ correspond to the Aharonov–Bohm phase, vector potential and magnetic field, respectively. In this review, we focus on the k-space Berry curvature $\Omega_{n}(k)=\nabla_{k}\times A_{n}(k)=i\nabla_{k}\times \text{\textbackslash{}break}\;\left\langle u_{n}(k)|\nabla_{k}|u_{n}(k)\right\rangle$ as an origin of the large transverse responses in topological magnets, where $\left|u_{n}(k)\right\rangle$is the periodic part of the Bloch wave function $|\psi_{n,k}\rangle =e^{i\mathrm{kr}}|u_{n}(k)\rangle$.

## 4. Berry curvature driven intrinsic transverse thermoelectric effect

Similar to *σ*_*ij*_, *α*_*ij*_ can originate from both intrinsic and extrinsic mechanisms. Here, the term ‘intrinsic’ refers to the contribution from the *k*-space Berry curvature as discussed in Section 2. In MnBi, the experimentally observed *α*_*ij*_ is one order of magnitude larger than the theoretical value assuming the intrinsic mechanism, indicating large extrinsic contributions [57]. However, this is so far an exception; the intrinsic mechanism dominates in most of magnetic materials with large ANE. Thus, in this review, we focus on the intrinsic ANE driven by *k*-space Berry curvature. In this section, we first introduce the mechanism for the intrinsic AHE as it is related to the origin of the intrinsic ANE.

### 4.1. Intrinsic anomalous Hall conductivity

Using Kubo formula, we obtain [58,59]

(16) $$\begin{array}{c} \sigma_{\mathrm{xy}}\left(T,\mu \right)=-\frac{e^{2}}{\hbar }\int \frac{d^{D}k}{{\left(2\pi \right)}^{D}}\underset{n}{\sum }f_{nk}\Omega_{n}^{z}\left(k\right) \end{array},$$

where $D\left(=\;2,3\right)$, e, ℏ, μ, $\varepsilon_{nk}$ and $f_{nk}=1/(e^{(\varepsilon_{nk}-\mu )/k_{B}T}+1)$ are the spatial dimension of the system, elementary charge, reduced Planck constant, chemical potential, energy for the band *n*, and the Fermi-Dirac distribution function, respectively. Equation (16) yields the quantum Hall effect for 2D-band insulator at T= 0

(17) $$\sigma_{xy}^{QHE}=-\frac{e^{2}}{\hbar }\int_{\mathrm{BZ}}\frac{d^{2}k}{{\left(2\pi \right)}^{2}}\sum_{n}^{\mathrm{occupied}}\Omega_{n}^{z}(k)=-\frac{e^{2}}{\hbar }C,$$

where *C* = 1,2,3··· is the Chern number and matches with the number of occupied states [60]. Thus, the intrinsic AHE in 3D metals can be viewed as an ”unquantized” version of the quantum Hall effect. The Berry curvature Ω^*z*^_*n*_(**k**) in Eq. (16) is numerically computed by using

(18) $$\mathrm{\Omega nz}(k)=-2\text{Im}\sum m\neq n\langle u_{n}(k)|\frac{\partial H(k)}{\partial k_{x}}|u_{m}(k)\langle u_{m}(k)|\frac{\partial H(k)}{\partial k_{y}}|u_{n}(k){\left(\varepsilon_{mk}-\varepsilon_{nk}\right)}^{2},$$

which means that the magnitude of the Berry curvature $\left|\Omega_{n}^{z}\left(k\right)\right|$ becomes singularly large when two bands touch. This explains why topological magnets with band crossings (Figure 4 and Section 5) show a large AHE.

In ferromagnets, the spontaneous time-reversal symmetry breaking ensures a finite *σ*_*xy*_. By contrast, antiferromagnets require additional constraints for the non-vanishing AHE, i.e. *σ*_*xy*_ = 0 for the simplest collinear anitiferromagnets, where the spin structure is invariant time-reversal + half-unit cell translation [28,61,62]. Examples include Mn_3_*X* (*X* = Sn, Ge) as shown in Section 6.

### 4.2. Intrinsic transverse thermoelectric conductivity

Similar to $\sigma_{\mathrm{xy}}$, the transverse thermoelectric conductivity $\alpha_{\mathrm{xy}}$ also arises from the intrinsic Berry curvature mechanism [63], i.e.

(19) $$\alpha_{\mathrm{xy}}(T,\mu )=\frac{|e|}{T\hbar }\int \frac{dk}{{\left(2\pi \right)}^{3}}\Omega_{n,z}(k)\{(\varepsilon_{nk}-\mu )f_{nk}+k_{B}T\log[1+e^{-(\varepsilon_{nk}-\mu )/k_{\beta }}]\}$$

(20) $$\;=\frac{1}{|e|}\int \mathrm{d\varepsilon}\sigma_{\mathrm{xy}}(0,\varepsilon )\frac{\varepsilon -\mu }{T}\left(-\frac{\mathrm{\partial f}}{\partial \varepsilon }\right)\;$$

(21) $$=-\frac{k_{B}}{|e|}\int \mathrm{d\varepsilon}\frac{\partial \sigma_{\mathrm{xy}}(T,\varepsilon )}{\partial \varepsilon }s(\varepsilon ,T),$$

where $s=-f\;\ln\left(f\right)-\left(1-f\right)\;\ln\left(1-f\right)$ is the entropy density and $\partial \sigma_{\mathrm{xy}}/\partial \varepsilon =\sum_{nk}\Omega_{n,z}(k)\delta (\varepsilon -\varepsilon_{nk})$. Using the Sommerfeld expansion we obtain the low-temperature asymptotic form called the Mott relation

(22) $$\alpha_{\mathrm{xy}}={\left.-\frac{\pi^{2}k_{B}^{2}T}{3|e|}\frac{\partial \sigma_{\mathrm{xy}}(0,\;\varepsilon )}{\partial \varepsilon }\right|}_{\varepsilon =E_{F}}.$$

**Figure 3.**
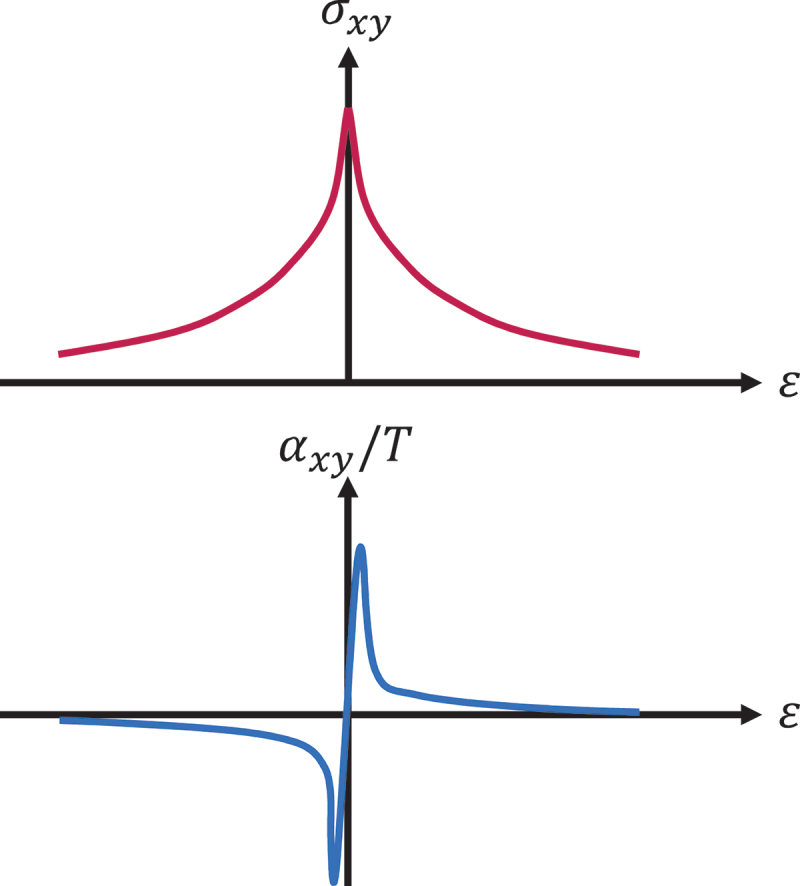
Schematic figures for the energy dependence of *σ*_*xy*_ (top) and *α*_*xy*_/*T* (bottom) at low temperatures, where the Mott relation (Eq. 22) holds [64]. Here, we assume topological band structure (e.g. Weyl points and nodal lines) inducing a large Berry curvature Ω_*z*_ at Fermi energy *ε* ∼ 0.

This implies that $|\alpha_{\mathrm{xy}}/T|$ becomes large when *σ*_*xy*_ varies rapidly as a function of *ε* owing to topological band structures (Figure 3) [64].

*σ*_*xy*_ and *α*_*xy*_ are often numerically computed using Eq. (16) and Eq. (19) by firstprinciples calculations, respectively. While $\sigma_{\mathrm{xy}}$ is given by the sum of the Berry curvature below *ϵ* ∼ *µ* (Eq. (16)), *α*_*xy*_ depends on the Berry curvature and density of states (DOS) near ε ∼ μ (Eq. 19). Thus, topological flat band structures that produce both large Berry curvature and DOS near the Fermi energy are essential for enhancing $\alpha_{\mathrm{xy}}$. Examples include the titled Weyl cones (Co_2_MnGa) [19], nodal web structure (Fe_3_Ga) [4,65], and nodal plane (Fe_3_Sn) [24], as discussed in Section 5.

## 5. Topological band structures inducing large Berry curvatures

**Figure 4.**
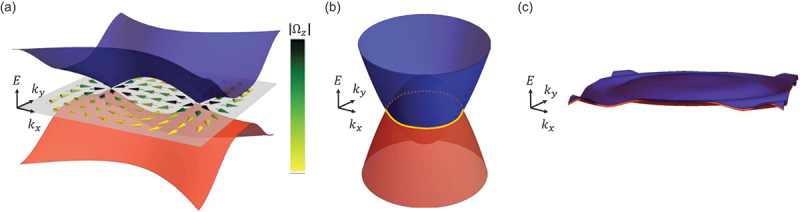
Schematic figures for topological band structures; (a) nodal points (Weyl points), (b) a nodal line and (c) a nodal plane. The arrows in (a) show the directions and magnitudes of the Berry curvature.

As discussed in Section 4, Berry curvature is highly enhanced near band crossings. Such band crossings occur at either (i) a k point (Weyl point, Figure 4(a), (ii) a line (nodal line, Figure 4(b), or (iii) a plane (nodal plane, Figure 4(c). The nodal line and plane are defined in the absence of the spin-orbit coupling (SOC). After introducing SOC, Weyl points may persist; otherwise, a small gap opens all over the nodal line (plane).

The Weyl point denotes a non-degenerate linear band crossing $\varepsilon_{k}\;∼\;\hbar v\left|k\pm k_{0}\right|$ and their model Hamiltonian is identical to the time-independent Weyl equation $H\psi_{k}\;∼\;\hbar v(k\pm k_{0})\sigma \psi_{k}=\varepsilon_{k}\psi_{k}$ [13,14,66]. In particular, non-vanishing AHE and ANE appear in magnetic Weyl semimetals (i.e. Weyl magnets), possessing Weyl points near the Fermi energy *ε*_F_ in a magnetically ordered state. Each Weyl point carries the chirality of ±1 and always pairs with the opposite signs [67]. These Weyl points act as either sources or sinks of the Berry curvature (Figure 4(a), inducing AHE and ANE.

## 6. Experimental realization – large ANE in topological magnets

Until now, various topological magnets have been discovered that exhibit large ANEs. In this section, we first describe the universal features shared across these materials, followed by details on specific compounds. The materials highlighted here are some the best-studied topological magnets, i.e. Mn_3_*X* (*X* = Sn, Ge) [16–18,22], Co_2_MnGa [19], Fe_3_*X* (*X* = Ga, Al) [4], YbMnBi_2_ [25,26], Fe_3_Sn [24], Co_3_Sn_2_S_2_ [20,21] and UCo_0.8_Ru_0.2_Al [23].

### 6.1. Dominant intrinsic contribution to σ_xy_ and α_xy_

The intrinsic Berry curvature contribution dominates both *σ*_*xy*_ and *α*_*xy*_ in topological magnets. For example, first-principles calculations that consider only the intrinsic contribution (Eq. 6 and Eq. 19) successfully reproduce the experimental *σ*_*xy*_ and *α*_*xy*_. The enhanced *σ*_*xy*_ and *α*_*xy*_ are associated with topological band structures, which are often confirmed by the ARPES (Angle-resolved Photoemission Spectroscopy) measurements. In most of these topological magnets, electron correlation effects are included only through the bandwidth renormalization, characterized by the ratio of Sommerfeld coefficient between experiment and theory, $\gamma_{\exp}/\gamma_{\mathrm{theory}}$.

**Figure 5.**
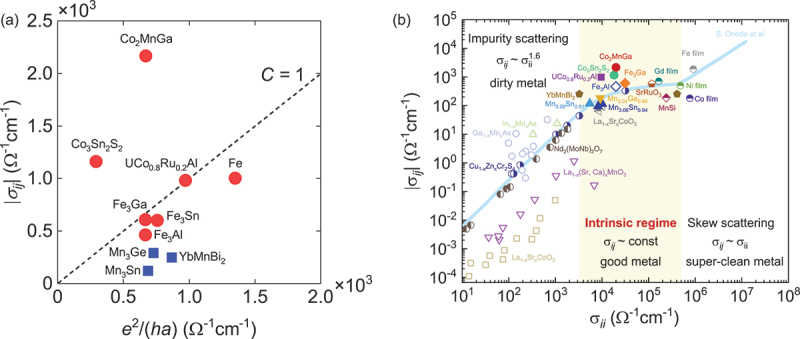
(a) The magnitude of the anomalous Hall conductivity |*σ*_*ij*_| compared to *e*^2^*/*(*ha*), where *a* is the lattice constant along the applied field direction; *a* = *b* = *c* = 5.77 Å for Co_2_MnGa, 5.80 Å for Fe_3_Ga, 5.79 Å for Fe_3_Al, 2.89 Å for Fe, *a* = *b* = 5.66 Å for Mn_3_Sn *a* = *b* = 5.33 Å for Mn_3_Ge, *a* = *b* = 4.47 Å for YbMnBi_2_, *c* = 13.18 Å for Co_3_Sn_2_S_2_, *c* = 3.97 Å for UCo_0.8_Ru_0.2_Al. for polycrystalline Fe_3_Sn, we used the averaged lattice constant (*a* + *b* + *c*)*/*3 ∼ 5.10 Å. (b) The scaling relation between |*σ*_*ij*_| and the longitudinal conductivity *σ*_*ii*_. Here, data taken at the lowest temperature (*T* ∼ 2 K) are used except for Mn_3_Sn (*T* ∼ 100 K). The references include refs [16, 17, 22] for Mn_3_Sn and Mn_3_Ge, ref [19] for Co_2_MnGa, ref [4] for Fe_3_Ga and Fe_3_Al, ref [26] for YbMnBi_2_, ref [24] for Fe_3_Sn, refs [20,21] for Co_3_Sn_2_S_2_, ref [23] for UCo_0.8_Ru_0.2_Al, ref [69] for Fe, Co, Ni and Gd thin films, ref [70] for MnSi, ref [68] for perovskite oxides (SrRuO_3_, La_1−*x*_Sr_*x*_CoO_3_, La_1−*x*_(Sr,Ca)_*x*_MnO_3_), spinels (Cu_1−*x*_Zn_*x*_Cr_2_Se_4_), pyrochlore (Nd_2_(MoNb)_2_O_7_) and magnetic semiconductor (Ga_1−*x*_Mn_*x*_As, In_1−*x*_Mn_*x*_As).

Another piece of evidence supporting the intrinsic contribution is the scaling relation between the anomalous Hall conductivity $\sigma_{\mathrm{xy}}$ and longitudinal conductivity *σ*_*xx*_ [68]. According to the scaling relation, *σ*_*xy*_ is almost constant and reaches $∼e^{2}/\left(\mathrm{ha}\right)∼10^{3}$ Ω^−1^cm^−1^ in the good metal regime $\sigma_{\mathrm{xx}}∼3\times 10^{3}$ $\Omega^{-1}$cm${\;}^{-1}$ - $5\times 10^{5}$ $\Omega^{-1}$cm${\;}^{-1}$ [68]. As shown in Figure 5(a,b), the topological magnets exhibit $\left|\sigma_{\mathrm{ij}}\right|∼e^{2}/\left(\mathrm{ha}\right)$ and satisfy these conditions.

### 6.2. Relation between ANE and magnetization

As mentioned above, the intrinsic *σ*_*xy*_ and *α*_*xy*_ originate from the *k*-space Berry curvature and thus they do not necessarily scale with the saturation magnetization. However, this is typically observed only at low *T*(where *M* is nearly saturated) and at high *B* (single-domain state). In other words, at high *T* and low *B*, ANE and AHE are usually related to the magnetization.

#### 6.2.1. Temperature dependence (Figure 6)

**Figure 7.**
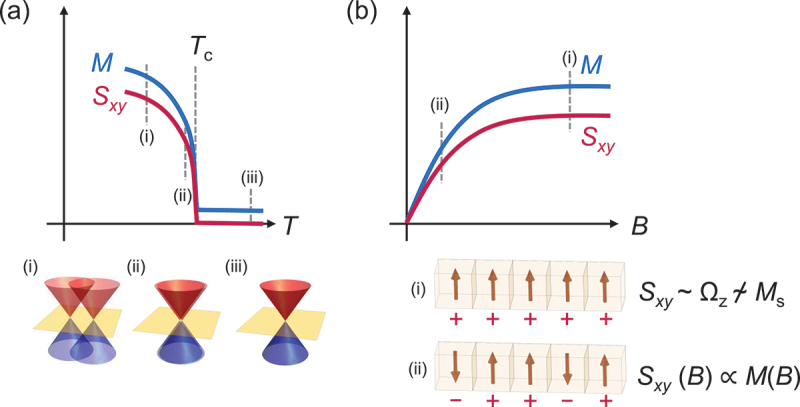
(a) Schematic figures for the *T* dependences of *M* and *S*_*xy*_ near the Curie temperature *T*_C_ (top), and Weyl (or Dirac) points for (i)-(iii). (b) Schematic figures for the *B* dependences of *M* and *S*_*xy*_ (top), magnetic domains (boxes & red vectors) and their contributions to *S*_*xy*_ (signs below boxes). The total *M* and *S*_*xy*_ are obtained by summing over each domain contribution (red vectors and signs, respectively). At high field (e.g. state at (i)), all the magnetic moments are parallel to the applied external field, and thus total *S*_*xy*_ reflects the Berry curvature Ω_*z*_, leading to the violation of the empirical scaling relation between the *magnitude* of *S*_*xy*_ and saturation magnetization *M*_*s*_. on the other hand, the *shape* of the *B* sweep curves are similar to each other; *S*_*xy*_(*B*) ∝ *M*(*B*). This is because both the total *S*_*xy*_(*B*) and *M*(*B*) are determined by integrating all the magnetic-domain contributions at low fields (e.g. the state at (ii)). Here, we assume the Ising anisotropy and *B* applied to the easy axis.

The AHE and ANE vanish above Curier temperature *T*_C_ (or Néel temperature *T*_N_) since they require spin-split band structure due to the time-reversal symmetry breaking. For instance, in Weyl magnets, Weyl points with opposite chirality annihilate into a four-fold degenerate Dirac point above *T*_C,N_. On cooling below *T*_C,N_, local magnetic moments ⟨*m*⟩ develop and induce spin splitting in the band structure. The size of ⟨*m*⟩ (⟨*m*⟩ ∝ *M* for ferromagnets) is usually a good indicator for the magnitude of spin-splitting (and distance between Weyl points for Weyl magnets). Thus *T* dependence of ANE and AHE resembles that for *M* near *T*_C,N_. In particular, this is evident for low *T*_C,N_ materials such as YbMnBi_2_, Co_3_Sn_2_S_2_, and UCo_0.8_Ru_0.2_Al (Figure 7) [21,23,25,26]. Since the first-principles calculations normally fix the moment size ⟨*m*⟩, a comparison to the experiment should be performed at *T* ≪ *T*_C_, where the magnetization becomes nearly *T* independent.

#### 6.2.2. Magnetic field dependence (Figure 7(b))

At high *B*, the Berry curvature Ω_*z*_ dominates *σ*_*xy*_ and *α*_*xy*_, leading to the violation of the empirical scaling relation with the saturation magnetization *M*_*s*_. On the other hand, at low *B*, the curves of *S*_*xy*_(*B*) and *ρ*_*xy*_(*B*) resemble that of *M*(*B*), as magnetic domains are not fully aligned. Namely, integrating the contributions from all the magnetic domains that have either parallel or antiparallel *M* to the magnetic field direction, *S*_*xy*_ and *ρ*_*xy*_ are generally proportional to *M* since the sign of each domain’s contribution depends on its magnetization direction (Figure 7(b)).

The zero-field spontaneous ANE is important for thermoelectric applications. This occurs in hard magnets with large magnetocrystalline anisotropy *K*_*u*_ [J/m^3^] and small saturation magnetization *M*_*s*_ [A/m], i.e. larger magnetic hardness parameter *κ*_h_ than unit; $\kappa_{h}=\sqrt{\frac{K_{u}}{\mu_{0}M_{s}^{2}}}>1$ [71]. For soft magnets such as Co_2_MnGa, Fe_3_*X* (*X* = Ga, Al), and Fe_3_Sn shown below, zero-field spontaneous ANE occurs only in thin-film form under perpendicular temperature gradient to the film plane since the shape anisotropy stabiles the in-plane magnetization.

### 6.3. Diverging behavior of α_xy_/T (Figure 8)

**Figure 8.**
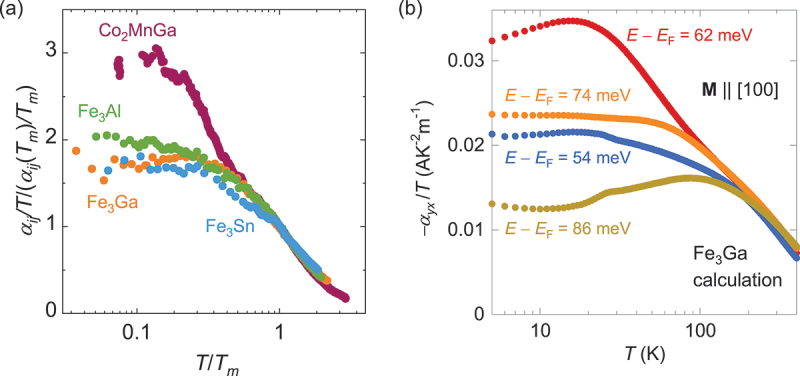
(a) The scaling relation of the transverse thermoelectric coefficient, *α*_*ij*_*/T/*(*α*_*ij*_(*T*_*m*_)*/T*_*m*_) vs *T/T*_*m*_, for Co_2_MnGa [19], Fe_3_*X* (*X* = Ga, Al) [4] and Fe_3_Sn [24]. Here, *T*_*m*_ is the temperature where *α*_*ij*_ shows maximum. (b) *T* dependence of *α*_*xy*_*/T* at various energy *E* −*E*_F_ obtained from the first-principles calculation for Fe_3_Ga. as the energy associated with the topological flat band approaches the Fermi energy, *α*_*xy*_ shows −ln*T* divergence down to much lower temperatures.

At low *T*, *α*_*xy*_*/T* should be constant as expected from the Mott relation (Eq 22), and finally *α*_*xy*_ = 0 at *T* = 0. On the other hand, various topological magnets show *α*_*xy*_*/T* ∼ −ln *T* at high *T* (Figure 8(a)). This logarithmic enhancement arises from the anomalous enhancement of *σ*_*xy*_(*ϵ*) and *α*_*xy*_(*ϵ*) due to the topological flat band structure (Section 4 & Figure 3), which is also reproduced by the first-principles calculations for Co_2_MnGa and Fe_3_Ga (Figure 8(b)) [4,19,65]. As the topological flat band approaches the Fermi energy *E*_F_, *α*_*xy*_ shows −ln*T* divergence to lower temperatures (Figure 8(b)).

#### 6.3.1. Mn_3_X (X = Sn, Ge)

The realization of the spontaneous AHE without net magnetization has been a great challenge in condensed matter physics. It was first discovered in the chiral spin liquid state of Pr_2_Ir_2_O_7_ at low temperatures [50] and later found in the inverse-triangular spin state of Mn_3_Sn at room temperature [16]. Mn_3_*X* (*X* = Sn, Ge) has a hexagonal Ni_3_Sn-type (*D*0_19_) crystal structure (space group *P*6_3_*/mmc*), where Mn atoms form breathing-type kagome layers in the *ab* plane [72]. Mn_3_*X* exhibits inverse-triangular non-collinear antiferromagnetic ordering at *T*_N_ ∼ 430 K (*X* = Sn) and 372 K (*X* = Ge) (Table 1) with a vanishingly small net spontaneous magnetization of several m*µ*_B_ in *ab* plane [83–86]. Nevertheless, Mn_3_*X* shows a large spontaneous Hall effect [16,87,88], Nernst effect [17,18,22] and magneto-optical Kerr effect [22,89,90] comparable to those for ferromagnets. Besides, Mn_3_*X* also exhibits spintronic phenomena such as the magnetic spin Hall effect [91], electric switching of AFM domains [92–94] and tunneling magnetoresistance [95]. These interesting properties are intimately linked to the magnetic structure and *k*-space topology: (1) The magnetic point group symmetry *m*^′^*m*^′^*m* for Mn_3_Sn is compatible with ferromagnetism, which allows the finite spontaneous AHE and ANE. This situation can be naturally interpreted by considering the magnetic structure as a cluster multipole [62,96]. (2) The ARPES measurements on Mn_3_Sn reveal Weyl points around K point near *E*_F_, consistent with the first-principles calculations [97]. These Weyl points act as sources or sinks of the Berry curvature, enhancing the AHE and ANE.

**Table 1.** Crystal structure and spin texture, type of magnetic order (FM or AFM), magnetic ordering temperature (*T*_C_ or *T*_N_), bandstructure topology, *S*_*xy*_ at room temperature, and related references for various topological magnets showing the large ANE.

Material	Crystal structure an spin texture	FM or AFM	*T*_C_ or *T*_N_ (K)	Band structure topology	*Sxy* at room *T* (*µ*V/K)	Ref.
Mn_3_*X* (*X* = Sn, Ge)	Hexagonal Ni_3_Sn-type (D0_19_) 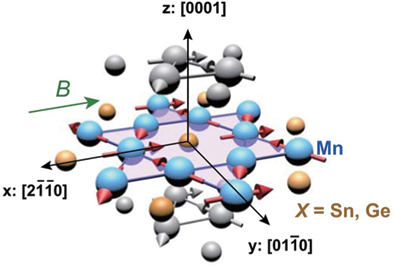 Adapted from [22]	AFM	430 (*X* = Sn), 372 (*X* = Ge)	Weyl points	∼−0.5	ANE [17,18,22] AHE [16] Device [31] Review [8,22,27,28]
Co_2_MnGa, Co_2_MnAl_1_−*x*Si_*x*_	Cubic full Heusler (*L*2_1_) 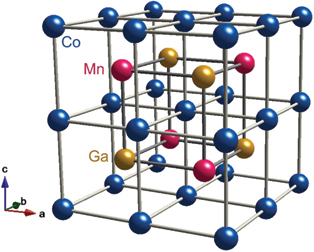	FM	694	Weyl points	∼8	ANE & AHE [19,73–76], Device [7], Review [8]
Fe_3_*X* (*X* = Ga, Al)	BCC derivative structure (*D*0_3_) 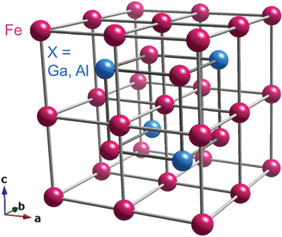	FM	720 (*X* = Al), 600 (*X* = Ga)	Nodal lines	∼4 (*X* = Al) ∼6 (*X* = Ga)	ANE [4,77–79], AHE, Device [30,33,34], Review [8]
YbMnBi_2_	tetragonal 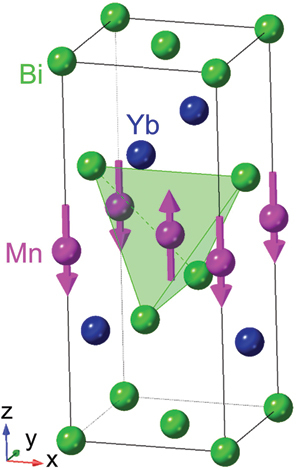 Adapted from [26]	AFM	∼290	Weyl points	0 (∼10 *µ*V/K at *T* ∼200 K)	AHE & ANE [25,26]
Fe_3_Sn	hexagonal Ni_3_Sn-type (D0_19_) 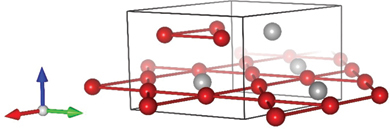 Adapted from [24]	FM	760	Nodal plane	∼3	AHE & ANE [24,80,81]
Co3Sn2S2	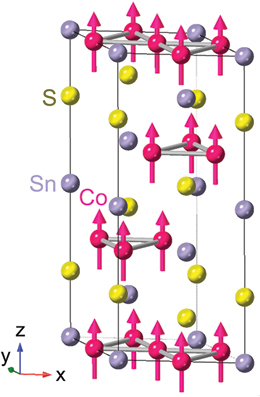 rhombohedral Adapted from [20]	FM	177	Weyl points	0 (∼3 *µ*V/K at *T* ∼75 K)	ANE [21], AHE [20]
UCo_0_.8Ru_0_.2Al	hexagonal ZrNiAl-type 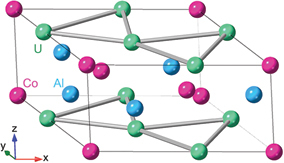 Adapted from [23]	FM 27	56	Weyl points	0 (∼23 *µ*V/K at *T* ∼40 K)	AHE [82] ANE [23]

Mn_3_*X* has been intensively studied for developing antiferromagnetic spintronics devices such as low-power and ultrafast nonvolatile memory [98–100]. For thermoelectric applications, the absence of stray fields and shape anisotropy in randomly oriented polycrystalline films has proven better for designing a highly dense array of the spintronic thermopiles with high heat flux sensitivity, compared to the conventional sensors based on ferromagnets [31].

#### 6.3.2. Co_2_MnGa, Co_2_MnAl_1−x_Si_x_

**Figure 6.**
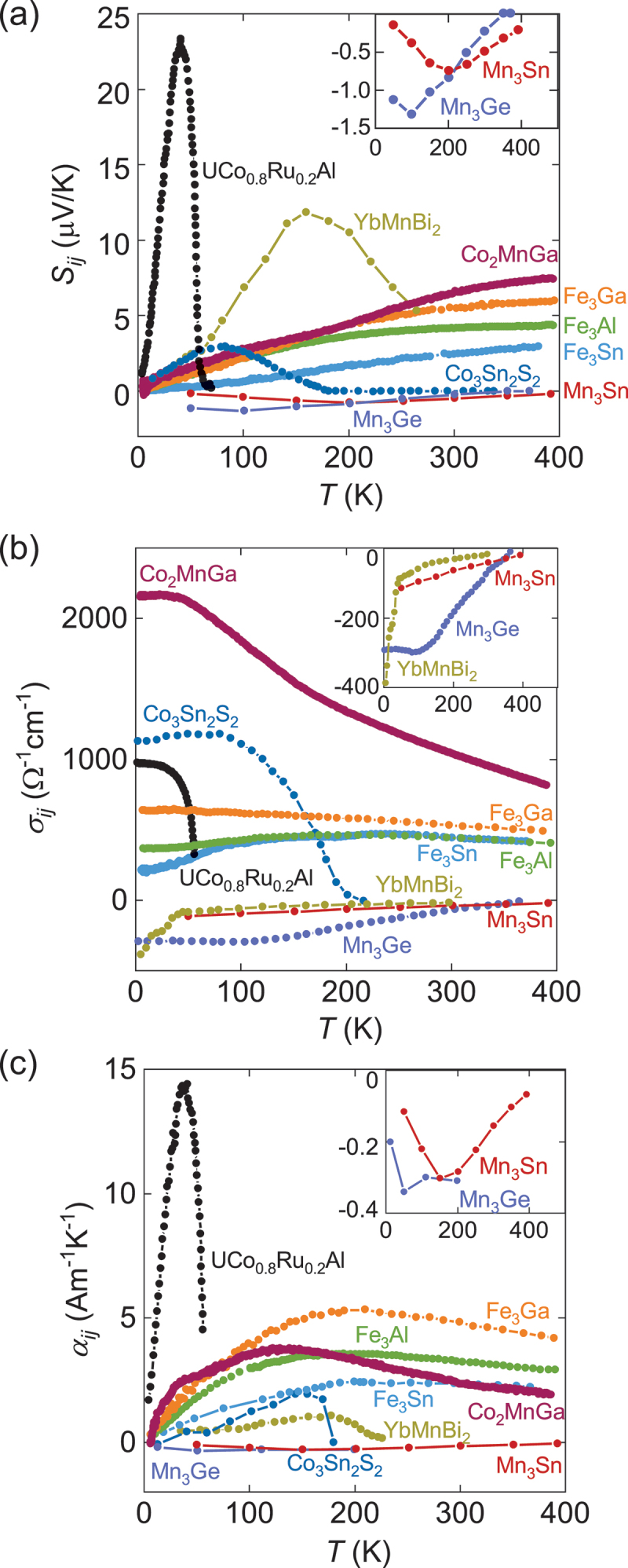
Temperature dependence of the anomalous Nernst coefficient *S*_*ij*_ (a), anomalous Hall conductivity *σ*_*ij*_ (b) and transverse thermoelectric conductivity *α*_*ij*_ (c) for various topological magnets. The measurement conditions, including the directions of electric current (**I**) and heat current (**Q**), are as follows: Mn_3_Sn (Mn_3.06_Sn_0.94_, *ij* = *zx*, **B** ∥ [0$\overset{ˉ}{1}$10], **I**, **Q** ∥ [$\overset{ˉ}{2}$$\overset{ˉ}{1}$10]) [17], Mn_3_Ge (Mn_3.03_Ge_0.97_, *ij* = *zx*, **B** ∥ [0$\overset{ˉ}{1}$10], **I**, **Q** ∥ [$\overset{ˉ}{2}$$\overset{ˉ}{1}$10]) [22], Co_2_MnGa (*ij* = *xy*, **B** ∥ [101], **I**, **Q** ∥ [001]) [19], Fe_3_*X* (*X* = Ga, Al) (*ij* = *xy*, **B** ∥ [101], **I**, **Q** ∥ [001]) [4], polycrystalline Fe_3_Sn [24], Co_3_Sn_2_S_2_ (**B** ∥ [0001], **I**, **Q** ∥ *ab* plane) [20,21], UCo_0.8_Ru_0.2_Al (**B** ∥ **c**, **I**, **Q** ∥ **a**) [23] and YbMnBi_2_ (−*S*_*zy*_, **B** ∥ **x**, **I**, **Q** ∥ **z**) [26]. Insets show zoomed-in region where the *y* axis is negative.

**Figure 9.**
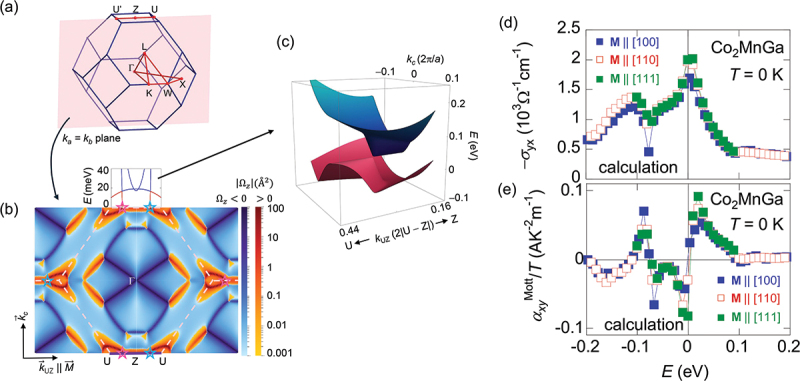
Topological band structure and large transverse responses obtained from the first-principles calculations for Co_2_MnGa (adapted from [19]). (a) First brillouin zone with *k*_*a*_ = *k*_*b*_ plane highlighted (pink). (b) Berry curvature on *k*_*a*_ = *k*_*b*_ plane. (c) Tilted Weyl cone near the Z-U line. (d, e) calculated anomalous Hall conductivity −*σ*_*yx*_ (d) and transverse thermoelectric conductivity *α*_*xy*_/*T* (e). Here *α*_*xy*_/*T* is obtained from the Mott relation (Eq. 22).

The above-mentioned ANE for Mn_3_Sn is surprisingly large for AFM and comparable to that in ferromagnets. However, its magnitude is still small |*S*_*xy*_| ∼ 0.5 *µ*V/K at room *T*. A much large ANE |*S*_*xy*_| ∼ 6–8 *µ*V/K, over an order of magnitude larger than in conventional magnets, was first reported in the Weyl ferromagnet Co_2_MnGa [19,73,74,76]. This remains the largest room-*T* value reported to date (Figure 6(a)). The crystal structure of Co_2_MnGa is the *L*2_1_ ordered cubic full Heusler (space group *Fm*$\overset{ˉ}{3}$*m*) and the Curie temperature is *T*_C_ ∼ 694 K (Table 1) [102]. The Hall conductivity *σ*_*xy*_ gradually increases on cooling and reaches *σ*_*xy*_ ∼ 2000 Ω^−1^cm^−1^ (Figure 6(b)), consistent with the first-principles calcinations. The transverse thermoelectric conductivity *α*_*xy*_ for Co_2_MnGa first increases on cooling, peaks at ∼150 K, and finally vanishes at low *T* (Figure 6(c)), again consistent with the first-principles calculations after considering the renormalization factor due to the correlation effect. The first-principles calculations attribute the large transverse responses to the enhanced Berry curvature near the tilted Weyl cones (Figure 9), which ARPES measurements confirm later [103–106]. The tilted Weyl cone in Co_2_MnGa is interpreted as a Lifshitz transition between type-I and type-II Weyl semi-metallic states, where one of the bands becomes nearly flat at *E* ∼ *E*_F_. This topological flat band is the key for the large *α*_*xy*_ in Co_2_MnGa. The large transverse responses for Co_2_MnGa are also promising for thermoelectric and spintronics device applications [7,107,108].

To date, most thermoelectric applications of the ANE focus on heat flux sensors utilizing the large voltage in the thin-film thermopile structure. Since ANE materials are typically metallic with relatively high thermal conductivity, they are suitable for efficient heat flux sensing. However, power generation from waste heat remains a significant challenge. A problem is the low conversion efficiency $\eta_{\mathrm{ANE}}=\eta_{C}\frac{1-\sqrt{1-Z_{\mathrm{yx}}T_{\mathrm{av}}}}{1+\sqrt{1-Z_{\mathrm{yx}}T_{\mathrm{av}}}}$ due to the small figure of merit $Z_{\mathrm{yx}}T=\sigma_{\mathrm{xx}}S_{yx}^{2}T/\kappa_{\mathrm{yy}}$. For example, even for Co_2_MnGa, the ANE material with the largest $S_{\mathrm{yx}}$ at room T, $Z_{\mathrm{yx}}T∼0.0001$ [19] partly due to the high thermal conductivity. Here, $T_{\mathrm{av}}=(T_{h}+T_{c})/2$ and $\eta_{C}=(T_{h}-T_{c})/T_{h}$ are the average *T* between high *T*_h_ and low *T*_c_ temperatures, and Carnot efficiency, respectively [101]. We note that $\eta_{\mathrm{ANE}}\to \eta_{C}$ at $Z_{\mathrm{yx}}T\to 1$, which is different from the Seebeck effect case, where $\eta_{\mathrm{SE}}=\eta_{C}\frac{\sqrt{1+ZT_{\mathrm{av}}}-1}{\sqrt{1+ZT_{\mathrm{av}}}+T_{c}/T_{h}}\to \eta_{C}$ at $\mathrm{ZT}=\sigma_{\mathrm{xx}}S_{xx}^{2}T/\kappa_{\mathrm{xx}}\to \infty$.

When considering applications, material cost is also an important factor. In the case of Co_2_MnGa, Ga is rather expensive compared to Co and Mn. Although the ANE *S*_*xy*_ ∼ 1 *µ*V/K for pure Co_2_MnAl is not so large as 6 - 8 *µ*V/K in Co_2_MnGa, the Si-doping enhances the ANE upto *S*_*xy*_ ∼ 5.7 *µ*V/K in Co_2_MnAl_0.63_Si_0.37_ [75]. Since both Al and Si are low-cost and naturally abundant elements, the system is promising for industrial applications.

#### 6.3.3. Fe_3_X (X = Ga, Al)

**Figure 10.**
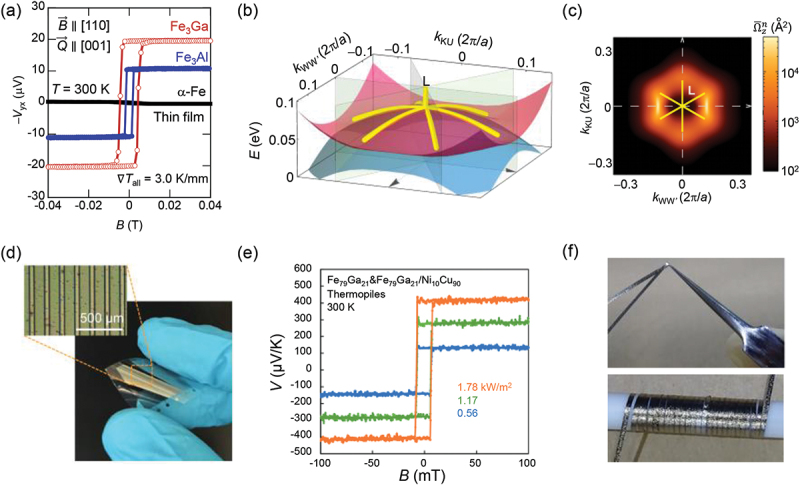
(a) Anomalous Nernst voltages for Fe_3_Ga and Fe_3_Al epitaxial thin films compared to that for *α*-Fe. Heat current was applied perpendicular to the film **Q** ∥ [001] and with in-plane magnetic field, **B** ∥ [001]. (b), (c) Topological band structure (nodal web) (b) and Berry curvature (c) near the L point obtained from the first-principles calculations for Fe_3_*X*. ((a)–(c) adapted from [4]) (d) Flexible Fe_3_Ga-based heat flux sensor fabricated on PET (polyethylene terephthalate) substrate. (e) *B* dependence of the ANE voltage for the Fe_3_Ga-based flexible heat flux sensor. Here, in-plane Seebeck effect is compensated by using Ni_10_Cu_90_ as electrodes since *S*_*ii*,Co2MnGa_ ∼ −*S*_*ii*,Ni10Cu90_. ((d), (e) adapted from [33]). (d) The ribbon-shaped Fe_3_Al (Adapted from [34]).

The discovery of the large ANE in Co_2_MnGa has stimulated the study of thermoelectric applications based on ANE. In addition to the large ANE, low material cost is an important factor for such applications. Iron is abundant and inexpensive, but the ANE for body-centered cubic (bcc) iron (*α*-Fe) is very small (|*S*_*xy*_| ∼ 0.3 *µ*V/K). With the help of high-throughput first-principles calculations, a large ANE has been identified in Fe_3_*X* (*X* = Ga, Al) [4]. Fe_3_*X* (*X* = Ga, Al) crystallizes in a bcc *D*0_3_ structure and shows ferromagnetic order below *T*_C_ ∼ 720 K (Fe_3_Ga) and ∼ 600 K (Fe_3_Al) [109,110]. As shown in Figure 6(a), Fe_3_*X* exhibits a large ANE −*S*_*yx*_ ∼ 5.7 *µ*V/K (Fe_3_Ga) and ∼ 4.0 *µ*V/K (Fe_3_Al) at room temperature [4], which is more than an order of magnitude larger than that for bcc iron [111]. The large spontaneous ANE is also confirmed in epitaxial thin films (Figure 10(a)). Besides, the room-*T α*_*xy*_ is also very large *α*_*xy*_ ∼ 5 (Am^−1^K^−1^). The first-principles calculations reveal that a nodal web structure – multiple interconnected nodal lines – near the L point gives rise to large Berry curvatures (Figure 10(b,c) [4,65]. The large ANE in Fe_3_*X* is robust against chemical doing as found in Fe_*x*_Ga_4−*x*_ (3.07 * < x <*3.12) and Fe_3_Ga_1−*x*_Al_*x*_ (0 ≲ *x* ≲ 0.6) polycrystals [78,79], as well as in epitaxial Fe-Ga thin films without post-annealing [77], which are all found beneficial for thermoelectric applications. For example, flexible heat flux sensors have been fabricated with mass-producible roll-to-roll sputtering method (Figure 10(d,e)) [33]. Ribbon-shaped Fe_3_Al alloys have also been produced, which can be directly wrapped around heat pipes (Figure 10(f)) [34]. Fe_3_*X* is also promising for spintronics device applications due to the low Gilbert damping constant [112,113].

#### 6.3.4. YbMnBi_2_

Topological antiferromagnets are attractive even for thermoelectric applications in electronic devices owing to their large responses and small stray field. The noncollinear antiferromagnet YbMnBi_2_ provides a large ANE $-S_{\mathrm{zy}}∼10$ μV/K at T∼200 K (Figure 6(a)) [25,26]. YbMnBi_2_ has a primitive tetragonal lattice (nonsymmorphic space group *P*4*/nmm*) and shows nearly collinear (C-type) AFM along *c* axis with a small cant along *ab*-palne below *T*_N_ ∼ 280 K [25,26,114–116]. The electronic band structure for YbMnBi_2_ is highly anisotropic, resulting in highly anisotropic transport properties such as $\rho_{\mathrm{zz}}>\rho_{\mathrm{yy}}$, $S_{\mathrm{yy}}<0<S_{\mathrm{zz}}$ and $S_{\mathrm{yz}}\;\neq -S_{\mathrm{zx}}$. On the other hand, samples are highly sensitive to oxygen and easily change their properties in the air [26]. For instance, the violation of the Onsager relation for *σ*_*ji*_ reported in [25] may be caused by the sample degradation [26]. Thus, industrial applications using this material are currently not straightforward.

### 6.4. Fe_3_Sn

As discussed in Section 4, topological flat bands are key for enhancing the transverse thermoelectric coefficient. Kagome metals are good candidates since *s*-electrons on the kagome lattice are known to provide a flat band owing to the destructive interference [117,118]. The kagome ferromagnet Fe_3_Sn shares the same crystal structure as Mn_3_*X* (*X* = Sn, Ge), hexagonal Ni_3_Sn type (*D*0_19_, space group *P*6_3_*/mmc*), where Fe atoms form a kagome lattice in *ab* plane (Table 1) [119]. The Curie temperature for Fe_3_Sn is *T*_C_ ∼ 760 K, which is high among topological magnets (Table 1). The large ANE *S*_*ij*_ ∼ 3 *µ*V/K at room *T* in Fe_3_Sn was first reported in bulk polycrystals [24] and later in amorphous and epitaxial thin films [80,81]. As shown in Figure 6(a), *S*_*ij*_ for polycrystalline Fe_3_Sn increases linearly at 100 K≲ *T* ≲ 400 K. The first-principles calculations reveal that the large ANE for Fe_3_Sn originates from a sharp peak in *σ*_*yz*_ at *E* − *E*_F_ ∼ 50 meV, which comes from a nodal plane – nearly degenerate, partiallyflat bands – near the Brillouin zone boundary [24]. These partially flat bands might originate from the destructive interference in the kagome lattice. The topological ANE found in the thin film based on the amorphous form of Fe_3_Sn makes this material attractive for the thermoelectric applications.

### 6.5. Co_3_Sn_2_S_2_

**Figure 11.**
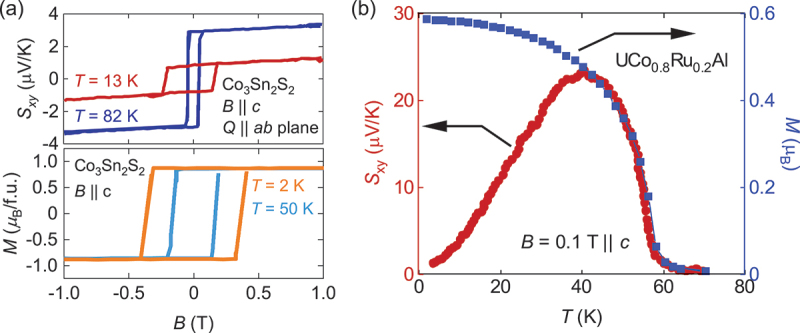
(a) *B* dependence of *S*_*xy*_ (top) and *M* (bottom) for Co_3_Sn_2_S_2_. The data are taken from ref [21]. The difference in coercivity between *S*_*xy*_ and *M* can be attributed to the difference in *T* and sample shape. (b) *T* dependence of *S*_*xy*_ (left axis) and *M* (right axis) for UCo_0.8_Ru_0.2_Al. The data are taken from ref [23].

A Co-based shandite Co_3_Sn_2_S_2_ is another kagome ferromagnet with *T*_C_ ∼ 177 K. It crystallizes in a rhombohedral structure (space group *R*$\overset{ˉ}{3}$*m*). The magnetic Weyl semimetal phase in Co_3_Sn_2_S_2_ is confirmed by various techniques, including ARPES (Fermi-arcs and linear bulk band crossings), STM (Scanning Tunneling Microscope) (quasi-particle interference), and magnetoresistance (chiral anomaly) [20,120]. Owing to the strong magnetocrystalline anisotropy and relatively small saturation magnetization *M*_*s*_ ∼ 0.8 *µ*_B_*/*f.u., the ANE shows clear hysteresis with the sizeable spontaneous value *S*_*xy*_ ∼ 3 *µ*V/K even in bulk form when *B* is applied along the easy axis *B* ∥ *c* (Figure 11) [21]. While the Curie temperature is lower than room *T*, these properties are attractive for thermoelectric applications at low *T*.

### 6.6. UCo_0.8_Ru_0.2_Al

Another approach for enhancing the DOS at the Fermi energy is via strong electronic correlations, especially in *f*-electron systems. The U-based ferromagnet UCo_0.8_Ru_0.2_Al shows the largest ANE *S*_*xy*_ ∼ 23 *µ*V/K (Figures 6(a) and 11(b)) and the transverse thermoelectric conductivity *α*_*ij*_ ∼ 14 Am^−1^K^−1^ at *T* ∼ 40 K (Figure 6(c)) [23]. The crystal structure is noncentrosymmetric hexagonal ZrNiAl-type (space group *P*¯$\overset{ˉ}{6}$2*m*) and U atoms form the distorted Kagome lattice in the *ab* plane (Table 1). The Sommerfeld coefficient for UCo_0.8_Ru_0.2_Al is *γ* ∼ 50 mJ/(mol K^2^), indicating moderately heavy charge carriers probably due to electronic correlations. The large *σ*_*xy*_ and *α*_*xy*_ for UCo_0.8_Ru_0.2_Al are reproduced by the first-principles calculations, indicating the large Berry curvature due to topological band structures. Indeed, the first-principles calculations find a large number of Weyl points (148 within *E* − *E*_F_ ∼ ±60 meV), which may contribute to enhancing the Berry curvature and *α*_*xy*_. Although this material is not suitable for applications due to the uranium and low Curie temperature *T*_C_ ∼ 56 K [82], this work provides valuable insight into further enhancement of the ANE.

## 7. Summary and perspective

Recent advances in topological condensed matter physics have led to the discovery of large ANEs in a variety of topological magnets, opening the way for their application at room temperature. Since the direction of the ANE-induced electromotive force is controlled by magnetization, even polycrystalline samples can generate strong signals [24,78,79], which is beneficial for industrial applications. ANE is better suited for flexible thin-wire circuit architectures, compared to the conventional Seebeck effect. Various ANE-based thermoelectric devices including high-sensitivity thin-film heat flow sensors have been fabricated, and some are fabricated by the mass-producible roll-to-roll sputtering method [33] and ready to be available commercially.

On the other hand, there is still room for improving their functionalities, in particular, for power generation. As discussed in Section 6, the magnitude of ANE is still much smaller than the Seebeck effect, resulting in a small figure of merit *Z*_*xy*_*T* ∼ 0.001 [19]. In addition to the band engineering relying on the intrinsic AHE, the extrinsic contributions such as magnon-driven ANE (e.g. MnBi [57]) would provide a new route for enhancing ANE. Another important aspect is the zero-field spontaneous ANE in bulk form. As already known in the field of permanent magnets [71], rare-earth magnets with large magnetocrystalline anisotropy would be suitable, though they are costlier. In addition to these functionalities, high Curie temperature, cost-effectiveness, and figure of merit in polycrystalline samples are important factors, while satisfying all the conditions would be rather challenging. Nevertheless, we believe that the innovative work reviewed in this article will inspire future research and uncover novel physical properties and functionalities in magnetic materials to improve the ANE.

## References

[1] Bell LE. Cooling, heating, generating power, and recovering waste heat with thermoelectric systems. Sci. 2008;321(5895):1457–17. doi: 10.1126/science.1158899 18787160

[2] Glatz W, Schwyter E, Durrer L, et al. Bi_2_Te_3_-based flexible micro thermoelectric generator with optimized design. J Microelectromech Syst. 2009 June;18(3):763–772. doi: 10.1109/JMEMS.2009.2021104

[3] Hu G, Edwards H, Lee M. Silicon integrated circuit thermoelectric generators with a high specific power generation capacity. Nat Electron. 2019;2(7):300–306. doi: 10.1038/s41928-019-0271-9

[4] Sakai A, Minami S, Koretsune T, et al. Iron-based binary ferromagnets for transverse thermoelectric conversion. Nature. 2020;581(7806):53–57. doi: 10.1038/s41586-020-2230-z 32376952

[5] Sakuraba Y, Hasegawa K, Mizuguchi M, et al. Anomalous Nernst effect in L10FePt/MnGa thermopiles for new thermoelectric applications. Appl Phys Express. 2013 mar;6(3):033003. doi: 10.7567/APEX.6.033003

[6] Mizuguchi M, Nakatsuji S. Energy-harvesting materials based on the anomalous Nernst effect. Sci Tech Adv Mater. 2019;20(1):262–275. doi: 10.1080/14686996.2019.1585143 PMC6442159 30956732

[7] Ki U, Zhou W, Sakuraba Y. Transverse thermoelectric generation using magnetic materials. Appl Phys Lett. 2021;118(14). doi: 10.1063/5.0046877

[8] Nakatsuji S, Arita R. Topological magnets: functions based on Berry phase and multipoles. Annu Rev Condens Matter Phys. 2022;13(1):119–142. doi: 10.1146/annurev-conmatphys-031620-103859

[9] Hasan MZ, Kane CL. Colloquium: topological insulators. Rev Mod Phys. 2010 Nov;82(4):3045–3067. doi: 10.1103/RevModPhys.82.3045

[10] Qi XL, Zhang SC. Topological insulators and superconductors. Rev Mod Phys. 2011 Oct;83(4):1057–1110. doi: 10.1103/RevModPhys.83.1057

[11] Ando Y. Topological insulator materials. J Phys Soc Jpn. 2013;82(10):102001. doi: 10.7566/JPSJ.82.102001

[12] Armitage N, Mele E, Vishwanath A. Weyl and Dirac semimetals in threedimensional solids. Rev Mod Phys. 2018;90(1):015001. doi: 10.1103/RevModPhys.90.015001

[13] Wan X, Turner AM, Vishwanath A, et al. Topological semimetal and Fermi-arc surface states in the electronic structure of pyrochlore iridates. Phys Rev B. 2011 May;83(20):205101. doi: 10.1103/PhysRevB.83.205101

[14] Burkov AA, Balents L. Weyl semimetal in a topological insulator multilayer. Phys Rev Lett. 2011 Sep;107(12):127205. doi: 10.1103/PhysRevLett.107.127205 22026796

[15] Fang C, Chen Y, Kee HY, et al. Topological nodal line semimetals with and without spin-orbital coupling. Phys Rev B. 2015 Aug;92(8):081201. doi: 10.1103/PhysRevB.92.081201

[16] Nakatsuji S, Kiyohara N, Higo T. Large anomalous Hall effect in a non-collinear antiferromagnet at room temperature. Nat. 2015;527(7577):212. doi: 10.1038/nature15723 26524519

[17] Ikhlas M, Tomita T, Koretsune T, et al. Large anomalous Nernst effect at room temperature in a chiral antiferromagnet. Nat Phys. 2017;13(11):1085–1090. doi: 10.1038/nphys4181

[18] Li X, Xu L, Ding L, et al. Anomalous Nernst and Righi-Leduc effects in Mn_3_Sn: berry curvature and entropy flow. Phys Rev Lett. 2017 Aug;119(5):056601. doi: 10.1103/PhysRevLett.119.056601 28949739

[19] Sakai A, Mizuta YP, Nugroho AA, et al. Giant anomalous Nernst effect and quantum-critical scaling in a ferromagnetic semimetal. Nat Phys. 2018;14(11):1119–1124. doi: 10.1038/s41567-018-0225-6

[20] Liu E, Sun Y, Kumar N, et al. Giant anomalous Hall effect in a ferromagnetic kagome-lattice semimetal. Nat Phys. 2018 Nov;14(11):1125–1131. doi: 10.1038/s41567-018-0234-5 30416534 PMC6217931

[21] Guin SN, Vir P, Zhang Y, et al. Zero-field Nernst effect in a ferromagnetic kagome-lattice Weyl-semimetal Co_3_Sn_2_S_2_. Adv Mater. 2019;31(25):1806622. doi: 10.1002/adma.201806622 31044469

[22] Chen T, Tomita T, Minami S, et al. Anomalous transport due to Weyl fermions in the chiral antiferromagnets Mn_3_X, X = Sn, Ge. Nat Commun. 2021 Jan;12(1):572. doi: 10.1038/s41467-020-20838-1 33495448 PMC7835387

[23] Asaba T, Ivanov V, Thomas S, et al. Colossal anomalous Nernst effect in a correlated noncentrosymmetric kagome ferromagnet. Sci Adv. 2021;7(13):eabf1467. doi: 10.1126/sciadv.abf1467 33771869 PMC7997519

[24] Chen T, Minami S, Sakai A, et al. Large anomalous Nernst effect and nodal plane in an iron-based kagome ferromagnet. Sci Adv. 2022;8(2):eabk1480. doi: 10.1126/sciadv.abk1480 35030028 PMC8759748

[25] Pan Y, Le C, He B, et al. Giant anomalous Nernst signal in the antiferromagnet YbMnBi_2_. Nat Mater. 2022;21(2):203–209. doi: 10.1038/s41563-021-01149-2 34811495 PMC8810386

[26] Guo X, Li X, Zhu Z, et al. Onsager reciprocal relation between anomalous transverse coefficients of an anisotropic antiferromagnet. Phys Rev Lett. 2023 Dec;131(24):246302. doi: 10.1103/PhysRevLett.131.246302 38181139

[27] Manna K, Sun Y, Muechler L, et al. Heusler, Weyl and Berry. Nat Rev Mater. 2018;3(8):244–256. doi: 10.1038/s41578-018-0036-5

[28] Šmejkal L, MacDonald AH, Sinova J, et al. Anomalous Hall antiferromagnets. Nat Rev Mater. 2022 Jun;7(6):482–496. doi: 10.1038/s41578-022-00430-3

[29] Narita H, Ikhlas M, Kimata M, et al. Anomalous Nernst effect in a microfabricated thermoelectric element made of chiral antiferromagnet Mn_3_Sn. Appl Phys Lett. 2017;111(20). doi: 10.1063/1.5000815

[30] Zhou W, Sakuraba Y. Heat flux sensing by anomalous Nernst effect in Fe–Al thin films on a flexible substrate. Appl Phys Express. 2020 mar;13(4):043001. doi: 10.35848/1882-0786/ab79fe

[31] Higo T, Li Y, Kondou K, et al. Omnidirectional control of large electrical output in a topological antiferromagnet. Adv functmater. 2021;31(15):2008971. doi: 10.1002/adfm.202008971

[32] Li X, Zhu Z, Behnia K. A monomaterial Nernst thermopile with hermaphroditic legs. Adv Mater. 2021;33(20):2100751. doi: 10.1002/adma.202100751 33844874

[33] Tanaka H, Higo T, Uesugi R, et al. Roll-to-roll printing of anomalous Nernst thermopile for direct sensing of perpendicular heat flux. Adv Mater. 2023;35(38):2303416. doi: 10.1002/adma.202303416 37343181

[34] Takigawa H, Taguchi T, Takahashi T, et al. Fabrication of thermoelectric ribbonshaped Fe_3_Al alloys with large anomalous Nernst effect. In: 2023 IEEE International Magnetic Conference (INTERMAG); 2023. Sendai, Japan: Sendai International Center. https://2023.intermag.org/

[35] Onsager L. Reciprocal relations in irreversible processes. Phys Rev. 1931 Feb;37(4):405–426. doi: 10.1103/PhysRev.37.405

[36] Onsager L. Reciprocal relations in irreversible processes. II. Phys Rev. 1931 Dec;38(12):2265–2279. doi: 10.1103/PhysRev.38.2265

[37] Callen HB. The application of Onsager’s reciprocal relations to thermoelectric, thermomagnetic, and galvanomagnetic effects. Phys Rev. 1948 Jun;73(11):1349–1358. doi: 10.1103/PhysRev.73.1349

[38] Hall EH. On a new action of the magnet on electric currents. Am J Math. 1879;2(3):287–292. doi: 10.2307/2369245

[39] Hall E. XVIII. On the “rotational coefficient” in nickel and cobalt. Lond Edinb Dublin Philos Mag J Sci. 1881;12(74):157–172. doi: 10.1080/14786448108627086

[40] Chien C. The Hall effect and its applications. New York, USA: Springer Science & Business Media; 2013.

[41] Fert A, Reyren N, Cros V. Magnetic skyrmions: advances in physics and potential applications. Nat Rev Mater. 2017 Jun;2(7):17031. doi: 10.1038/natrevmats.2017.31

[42] Tokura Y, Kanazawa N. Magnetic skyrmion materials. Chem Rev. 2021 Mar;121(5):2857–2897. doi: 10.1021/acs.chemrev.0c00297 33164494

[43] Rößler UK, Bogdanov AN, Pfleiderer C. Spontaneous skyrmion ground states in magnetic metals. Nat. 2006 Aug;442(7104):797–801. doi: 10.1038/nature05056 16915285

[44] Neubauer A, Pfleiderer C, Binz B, et al. Topological Hall effect in the *A* phase of MnSi. Phys Rev Lett. 2009 May;102(18):186602. doi: 10.1103/PhysRevLett.102.186602 19518895

[45] Yu XZ, Onose Y, Kanazawa N, et al. Real-space observation of a two-dimensional skyrmion crystal. Nat. 2010 Jun;465(7300):901–904. doi: 10.1038/nature09124 20559382

[46] Nagaosa N, Tokura Y. Topological properties and dynamics of magnetic skyrmions. Nat nanotechnol. 2013;8(12):899–911. doi: 10.1038/nnano.2013.243 24302027

[47] Fert A, Cros V, Sampaio J. Skyrmions on the track. Nat nanotechnol. 2013 Mar;8(3):152–156. doi: 10.1038/nnano.2013.29 23459548

[48] Shindou R, Nagaosa N. Orbital ferromagnetism and anomalous Hall effect in antiferromagnets on the distorted FCC lattice. Phys Rev Lett. 2001 Aug;87(11):116801. doi: 10.1103/PhysRevLett.87.116801 11531542

[49] Martin I, Batista CD. Itinerant electron-driven chiral magnetic ordering and spontaneous quantum Hall effect in triangular lattice models. Phys Rev Lett. 2008 Oct;101(15):156402. doi: 10.1103/PhysRevLett.101.156402 18999621

[50] Machida Y, Nakatsuji S, Onoda S, et al. Time-reversal symmetry breaking and spontaneous Hall effect without magnetic dipole order. Nat. 2010 Jan;463(7278):210–213. doi: 10.1038/nature08680 20010605

[51] Šmejkal L, Sinova J, Jungwirth T. Emerging research landscape of altermagnetism. Phys Rev X. 2022 Dec;12(4):040501. doi: 10.1103/PhysRevX.12.040501

[52] Jungwirth T, Sinova J, Fernandes RM, et al. Symmetry, microscopy and spectroscopy signatures of altermagnetism. arxiv Prepr arXiv: 250622860. 2025. doi: 10.48550/arXiv.2506.22860 41566009

[53] Ray MK, Fu M, Chen Y, et al. Zero-field Hall effect emerging from a non-Fermi liquid in a collinear antiferromagnet V_1*/*3_NbS_2_. Nat Commun. 2025;16(1):3532. doi: 10.1038/s41467-025-58476-0 40251162 PMC12008243

[54] Takagi R, Hirakida R, Settai Y, et al. Spontaneous Hall effect induced by collinear antiferromagnetic order at room temperature. Nat Mater. 2025;24(1):63–68. doi: 10.1038/s41563-024-02058-w 39672960

[55] Modak R, Sakuraba Y, Hirai T, et al. Sm-Co-based amorphous alloy films for zero-field operation of transverse thermoelectric generation. Sci Technol Adv Mater. 2022;23(1):767–782. doi: 10.1080/14686996.2022.2138538 36386550 PMC9662036

[56] Berry MV. Quantal phase factors accompanying adiabatic changes. Proc R Soc Of Lond A Math And Phys Sci. 1984;392(1802):45–57. doi: 10.1098/rspa.1984.0023

[57] He B, S¸ahin C, Boona SR, et al. Large magnon-induced anomalous Nernst conductivity in single-crystal MnBi. Joule. 2021;5(11):3057–3067. doi: 10.1016/j.joule.2021.08.007 34841198 PMC8604385

[58] Nagaosa N, Sinova J, Onoda S, et al. Anomalous Hall effect. Rev Mod Phys. 2010 May;82(2):1539–1592. doi: 10.1103/RevModPhys.82.1539

[59] Xiao D, Chang MC, Niu Q. Berry phase effects on electronic properties. Rev Mod Phys. 2010 Jul;82(3):1959–2007. doi: 10.1103/RevModPhys.82.1959

[60] Thouless DJ, Kohmoto M, Nightingale MP, et al. Quantized Hall conductance in a two-dimensional periodic potential. Phys Rev Lett. 1982 Aug;49(6):405–408. doi: 10.1103/PhysRevLett.49.405

[61] Chen H, Niu Q, MacDonald AH. Anomalous Hall effect arising from noncollinear antiferromagnetism. Phys Rev Lett. 2014 Jan;112(1):017205. doi: 10.1103/PhysRevLett.112.017205 24483927

[62] Suzuki MT, Koretsune T, Ochi M, et al. Cluster multipole theory for anomalous Hall effect in antiferromagnets. Phys Rev B. 2017 Mar;95(9):094406. doi: 10.1103/PhysRevB.95.094406

[63] Xiao D, Yao Y, Fang Z, et al. Berry-phase effect in anomalous thermoelectric transport. Phys Rev Lett. 2006 Jul;97(2):026603. doi: 10.1103/PhysRevLett.97.026603 16907470

[64] Sharma G, Goswami P, Tewari S. Nernst and magnetothermal conductivity in a lattice model of Weyl fermions. Phys Rev B. 2016 Jan;93(3):035116. doi: 10.1103/PhysRevB.93.035116

[65] Minami S, Ishii F, Hirayama M, et al. Enhancement of the transverse thermoelectric conductivity originating from stationary points in nodal lines. Phys Rev B. 2020 Nov;102(20):205128. doi: 10.1103/PhysRevB.102.205128

[66] Yang KY, Lu YM, Ran Y. Quantum Hall effects in a Weyl semimetal: possible application in pyrochlore iridates. Phys Rev B. 2011 Aug;84(7):075129. doi: 10.1103/PhysRevB.84.075129

[67] Nielsen H, Ninomiya M. Absence of neutrinos on a lattice: (i). Proof by homotopy theory. Nuc Phys B. 1981;185(1):20–40. doi: 10.1016/0550-3213(81)90361-8

[68] Onoda S, Sugimoto N, Nagaosa N. Quantum transport theory of anomalous electric, thermoelectric, and thermal Hall effects in ferromagnets. Phys Rev B. 2008 Apr;77(16):165103. doi: 10.1103/PhysRevB.77.165103

[69] Miyasato T, Abe N, Fujii T, et al. Crossover behavior of the anomalous Hall effect and anomalous Nernst effect in itinerant ferromagnets. Phys Rev Lett. 2007 Aug;99(8):086602. doi: 10.1103/PhysRevLett.99.086602 17930968

[70] Manyala N, Sidis Y, DiTusa JF, et al. Large anomalous Hall effect in a silicon-based magnetic semiconductor. Nat Mater. 2004 Apr;3(4):255–262. doi: 10.1038/nmat1103 15034565

[71] Coey J. Perspective and prospects for rare earth permanent magnets. Engineering. 2020;6(2):119–131. doi: 10.1016/j.eng.2018.11.034

[72] Krén E, Paitz J, Zimmer G, et al. Study of the magnetic phase transformation in the Mn_3_Sn phase. Physica B+C. 1975;80(1–4):226–230. doi: 10.1016/0378-4363(75)90066-2

[73] Guin SN, Manna K, Noky J, et al. Anomalous Nernst effect beyond the magnetization scaling relation in the ferromagnetic Heusler compound Co_2_MnGa. NPG Asia Mater. 2019 Apr;11(1):16. doi: 10.1038/s41427-019-0116-z

[74] Xu L, Li X, Ding L, et al. Anomalous transverse response of Co_2_MnGa and universality of the room-temperature *α_ij_^A^/σ_ij_^A^* ratio across topological magnets. Phys Rev B. 2020 May;101(18):180404. doi: 10.1103/PhysRevB.101.180404

[75] Sakuraba Y, Hyodo K, Sakuma A, et al. Giant anomalous nernst effect in the Co_2_MnAl_1−*x*_Si*_x_* heusler alloy induced by fermi level tuning and atomic ordering. Phys Rev B. 2020 Apr;101(13):134407. doi: 10.1103/PhysRevB.101.134407

[76] Uesugi R, Higo T, Nakatsuji S. Giant anomalous Nernst effect in polycrystalline thin films of the Weyl ferromagnet Co_2_MnGa. Appl Phys Lett. 2023;123(25). doi: 10.1063/5.0174663

[77] Nakayama H, Masuda K, Wang J, et al. Mechanism of strong enhancement of anomalous Nernst effect in Fe by Ga substitution. Phys Rev Mater. 2019;3(11):114412. doi: 10.1103/PhysRevMaterials.3.114412

[78] Feng Z, Minami S, Akamatsu S, et al. Giant and robust anomalous Nernst effect in a polycrystalline topological ferromagnet at room temperature. Adv Funct Mater. 2022;32(49):2206519. doi: 10.1002/adfm.202206519

[79] Wang Y, Sakai A, Minami S, et al. Robust giant anomalous Nernst effect in polycrystalline nodal web ferromagnets. Appl Phys Lett. 2024 08;125(8):081901. doi: 10.1063/5.0219416

[80] Fujiwara K, Kato Y, Abe H, et al. Berry curvature contributions of kagome lattice fragments in amorphous Fe–Sn thin films. Nat Commun. 2023 Jun;14(1):3399. doi: 10.1038/s41467-023-39112-1 37311774 PMC10264439

[81] Kurosawa S, Higo T, Saito S, et al. Large spontaneous magneto-thermoelectric effect in epitaxial thin films of the topological kagome ferromagnet Fe_3_Sn. Phys Rev Mater. 2024 May;8(5):054206. doi: 10.1103/PhysRevMaterials.8.054206

[82] Pospíšil J, Opletal P, Vališka M, et al. Properties and collapse of the ferromagnetism in UCo_1−*x*_Ru*_x_*Al studied in single crystals. J Phys Soc Jpn. 2016;85(3):034710. doi: 10.7566/JPSJ.85.034710

[83] Tomiyoshi S. Polarized neutron diffraction study of the spin structure of Mn_3_Sn. J Phy Soc Jpn. 1982;51(3):803–810. doi: 10.1143/JPSJ.51.803

[84] Brown PJ, Nunez V, Tasset F, et al. Determination of the magnetic structure of Mn_3_Sn using generalized neutron polarization analysis. J Phys Condens Matter. 1990 Nov;2(47):9409. doi: 10.1088/0953-8984/2/47/015

[85] Yamada N, Sakai H, Mori HY, et al. Magnetic properties of *ε*-Mn_3_Ge. Physica B+C. 1988;149(1–3):311–315. doi: 10.1016/0378-4363(88)90258-6

[86] Sukhanov AS, Singh S, Caron L, et al. Gradual pressure-induced change in the magnetic structure of the noncollinear antiferromagnet Mn_3_Ge. Phys Rev B. 2018 Jun;97(21):214402. doi: 10.1103/PhysRevB.97.214402

[87] Kiyohara N, Tomita T, Nakatsuji S. Giant anomalous Hall effect in the chiral antiferromagnet Mn_3_Ge. Phys Rev Appl. 2016 Jun;5(6):064009. doi: 10.1103/PhysRevApplied.5.064009

[88] Nayak AK, Fischer JE, Sun Y, et al. Large anomalous Hall effect driven by a nonvanishing Berry curvature in the noncolinear antiferromagnet Mn_3_Ge. Sci Adv. 2016;2(4):e1501870. doi: 10.1126/sciadv.1501870 27152355 PMC4846447

[89] Higo T, Man H, Gopman DB, et al. Large magneto-optical Kerr effect and imaging of magnetic octupole domains in an antiferromagnetic metal. Nat photon. 2018 Feb;12(2):73–78. doi: 10.1038/s41566-017-0086-z PMC5997294 29910828

[90] Matsuda T, Kanda N, Higo T, et al. Room-temperature terahertz anomalous Hall effect in Weyl antiferromagnet Mn_3_Sn thin films. Nat Commun. 2020 Feb;11(1):909. doi: 10.1038/s41467-020-14690-6 32060261 PMC7021706

[91] Kimata M, Chen H, Kondou K, et al. Magnetic and magnetic inverse spin Hall effects in a non-collinear antiferromagnet. Nat. 2019 Jan;565(7741):627–630. doi: 10.1038/s41586-018-0853-0 30651643

[92] Tsai H, Higo T, Kondou K, et al. Electrical manipulation of a topological antiferromagnetic state. Nat. 2020 Apr;580(7805):608–613. doi: 10.1038/s41586-020-2211-2 32350469

[93] Tsai H, Higo T, Kondou K, et al. Large Hall signal due to electrical switching of an antiferromagnetic Weyl semimetal state. Small Sci. 2021;1(5):2000025. doi: 10.1002/smsc.202000025 40212043 PMC11935791

[94] Higo T, Kondou K, Nomoto T, et al. Perpendicular full switching of chiral antiferromagnetic order by current. Nat. 2022 Jul;607(7919):474–479. doi: 10.1038/s41586-022-04864-1 35859198

[95] Chen X, Higo T, Tanaka K, et al. Octupole-driven magnetoresistance in an antiferromagnetic tunnel junction. Nat. 2023 Jan;613(7944):490–495. doi: 10.1038/s41586-022-05463-w PMC9849134 36653566

[96] Hayami S, Yanagi Y, Kusunose H. Bottom-up design of spin-split and reshaped electronic band structures in antiferromagnets without spin-orbit coupling: procedure on the basis of augmented multipoles. Phys Rev B. 2020 Oct;102(14):144441. doi: 10.1103/PhysRevB.102.144441

[97] Kuroda K, Tomita T, Suzuki MT, et al. Evidence for magnetic Weyl fermions in a correlated metal. Nat Mater. 2017 Nov;16(11):1090–1095. doi: 10.1038/nmat4987 28967918

[98] Jungwirth T, Marti X, Wadley P, et al. Antiferromagnetic spintronics. Nat nanotechnol. 2016 Mar;11(3):231–241. doi: 10.1038/nnano.2016.18 26936817

[99] Han J, Cheng R, Liu L, et al. Coherent antiferromagnetic spintronics. Nat Mater. 2023 Jun;22(6):684–695. doi: 10.1038/s41563-023-01492-6 36941390

[100] Rimmler BH, Pal B, Parkin SSP. Non-collinear antiferromagnetic spintronics. Nat Rev Mater. 2025 Feb;10(2):109–127. doi: 10.1038/s41578-024-00706-w

[101] Harman TC, Honig JM. Theory of galvano-thermomagnetic energy conversion devicesi. generators. J Appl Phys. 1962;33(11):3178–3188. doi: 10.1063/1.1931132

[102] Webster P. Magnetic and chemical order in Heusler alloys containing cobalt and manganese. J Phys Chem Solids. 1971;32(6):1221–1231. doi: 10.1016/S0022-3697(71)80180-4

[103] Belopolski I, Manna K, Sanchez DS, et al. Discovery of topological Weyl fermion lines and drumhead surface states in a room temperature magnet. Sci. 2019;365(6459):1278–1281. doi: 10.1126/science.aav2327 31604235

[104] Sumida K, Sakuraba Y, Masuda K, et al. Spin-polarized Weyl cones and giant anomalous Nernst effect in ferromagnetic Heusler films. Commun Mater. 2020 Nov;1(1):89. doi: 10.1038/s43246-020-00088-w

[105] Kono T, Kakoki M, Yoshikawa T, et al. Three-dimensional bulk Fermi surfaces and Weyl crossings of Co_2_MnGa thin films underneath a protection layer. Phys Rev B. 2021 Nov;104(19):195112. doi: 10.1103/PhysRevB.104.195112

[106] Belopolski I, Chang G, Cochran TA, et al. Observation of a linked-loop quantum state in a topological magnet. Nat. 2022 Apr;604(7907):647–652. doi: 10.1038/s41586-022-04512-8 35478239

[107] Leiva L, Granville S, Zhang Y, et al. Giant spin Hall angle in the Heusler alloy Weyl ferromagnet Co_2_MnGa. Phys Rev B. 2021 Jan;103(4):L041114. doi: 10.1103/PhysRevB.103.L041114

[108] Isshiki H, Zhu Z, Mizuno H, et al. Determination of spin Hall angle in the Weyl ferromagnet Co_2_MnGa by taking into account the thermoelectric contributions. Phys Rev Mater. 2022 Aug;6(8):084411. doi: 10.1103/PhysRevMaterials.6.084411

[109] Kawamiya N, Adachi K, Nakamura Y. Magnetic properties and M¨ossabauer investigations of Fe-Ga alloys. J Phys Soc Jpn. 1972;33(5):1318–1327. doi: 10.1143/JPSJ.33.1318

[110] Shinohara T. The effect of atomic ordering on the magnetic properties of Fe-Al alloys. J Phys Soc Jpn. 1964;19(1):51–58. doi: 10.1143/JPSJ.19.51

[111] Watzman SJ, Duine RA, Tserkovnyak Y, et al. Magnon-drag thermopower and Nernst coefficient in Fe, Co, and Ni. Phys Rev B. 2016;94(14):144407. doi: 10.1103/PhysRevB.94.144407

[112] Wei Y, Zhang W, Lv B, et al. Ultralow magnetic damping of a common metallic ferromagnetic film. Sci Adv. 2021;7(4):eabc5053. doi: 10.1126/sciadv.abc5053 33523919 PMC7817099

[113] Sakamoto S, Higo T, Tamaru S, et al. Low Gilbert damping in epitaxial thin films of the nodal-line semimetal *D*0_3_ Fe_3_Ga. Phys Rev B. 2021 Apr;103(16):165122. doi: 10.1103/PhysRevB.103.165122

[114] Wang A, Zaliznyak I, Ren W, et al. Magnetotransport study of Dirac fermions in YbMnBi_2_ antiferromagnet. Phys Rev B. 2016 Oct;94(16):165161. doi: 10.1103/PhysRevB.94.165161

[115] Borisenko S, Evtushinsky D, Gibson Q, et al. Time-reversal symmetry breaking type-II Weyl state in YbMnBi_2_. Nat Commun. 2019 Jul;10(1):3424. doi: 10.1038/s41467-019-11393-5 31366883 PMC6668437

[116] Soh JR, Jacobsen H, Ouladdiaf B, et al. Magnetic structure and excitations of the topological semimetal YbMnBi_2_. Phys Rev B. 2019 Oct;100(14):144431. doi: 10.1103/PhysRevB.100.144431

[117] Mielke A. Ferromagnetism in the Hubbard model on line graphs and further considerations. J Phys A: Math gen. 1991 Jul;24(14):3311. doi: 10.1088/0305-4470/24/14/018

[118] Tasaki H. Ferromagnetism in the Hubbard models with degenerate singleelectron ground states. Phys Rev Lett. 1992 Sep;69(10):1608–1611. doi: 10.1103/PhysRevLett.69.1608 10046265

[119] Sales BC, Saparov B, McGuire MA, et al. Ferromagnetism of Fe_3_Sn and alloys. Scic Rep. 2014 Nov;4(1):7024. doi: 10.1038/srep07024 PMC4228330 25387850

[120] Liu DF, Liang AJ, Liu EK, et al. Magnetic Weyl semimetal phase in a kagomé crystal. Sci. 2019;365(6459):1282–1285. doi: 10.1126/science.aav2873 31604236

